# Probing Type‐II Multiferroicity in Monolayer NiBr_2_

**DOI:** 10.1002/advs.77081

**Published:** 2026-08-13

**Authors:** Aleš Cahlík, Antti Karjasilta, Anshika Mishra, Robert Drost, Mohammad Amini, Javaria Arshad, Büşra Gamze Arslan, Peter Liljeroth

**Affiliations:** ^1^ Department of Applied Physics Aalto University Aalto Finland

**Keywords:** 2D materials, magnetoelectric coupling, monolayer, nickel dihalides, scanning tunneling microscopy, spin spiral, type II multiferroics

## Abstract

The recent discovery of type‐II multiferroicity in monolayer ${\mathrm{NiI}}_{2}$ indicated a new pathway for intrinsic magnetoelectric coupling in the two‐dimensional limit. However, whether this phenomenon is a unique feature of ${\mathrm{NiI}}_{2}$ or a chemically tunable property of the broader material class has remained unresolved. Here, we demonstrate that type‐II multiferroicity in the nickel dihalides is tunable through halide ligand substitution by visualizing the ferroelectric order in monolayer ${\mathrm{NiBr}}_{2}$. Using scanning tunneling microscopy (STM), we resolve atomic‐scale ferroelectric domains and confirm their magnetoelectric origin through reciprocal manipulation experiments: reorienting magnetic order via electric fields and suppressing the electric polarization with external magnetic fields. Furthermore, we find that the multiferroic state in ${\mathrm{NiBr}}_{2}$ is energetically less robust than in its iodide counterpart, consistent with modified superexchange interactions and the reduced spin‐orbit coupling (SOC). Our results establish the nickel dihalides as a versatile platform where the stability of magnetoelectric phases can be engineered through chemical substitution.

## Graphical abstract

Type‐II multiferroicity is demonstrated in monolayer NiBr_2_. Scanning tunneling microscopy resolves ferroelectric polarization arising from non‐collinear magnetic order, and reciprocal control with electric and magnetic fields confirms intrinsic magnetoelectric coupling. Comparison with NiI_2_ reveals how halide substitution governs the stability of the multiferroic state.

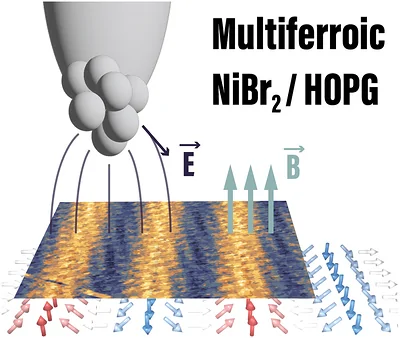

## 1 Introduction

A defining feature of two‐dimensional (2D) van der Waals materials is their chemical tunability [1]. Just as the electronic bandgap in transition metal dichalcogenides can be engineered by substituting the chalcogen atom (e.g., S, Se, Te), the physical properties of magnetic monolayers can be precisely tuned through ligand substitution [2, 3, 4]. This is exemplified in the transition metal dihalide (TMDH) family, where replacing the halogen ion (Cl, Br, I) systematically modifies the magnetic exchange interactions and spin‐orbit coupling (SOC) strength without altering the crystal symmetry [5, 6, 7, 8, 9]. This chemical simplicity offers a control knob to navigate the magnetic phase diagram, allowing researchers to stabilize and tune complex broken‐symmetry states in the monolayer limit.

One of the phases predicted to emerge from this tuning is “type‐II” multiferroicity. Type‐II multiferroicity is driven by a complex magnetic texture–typically a spin spiral–that spontaneously breaks spatial inversion symmetry [10, 11, 12, 13]. This mechanism is compelling because it inherently guarantees a strong, intrinsic coupling between the magnetic and electric order parameters. Such magnetoelectric interlocking allows for the manipulation of magnetic states via electric fields, a capability that is essential for the development of energy‐efficient spintronic devices [14, 15, 16]. Theoretical studies confirm that such non‐collinear ground states are intrinsic to the TMDH family [7, 17, 18, 19, 20, 21]. However, experimental realization in the monolayer limit remains exceptionally rare, as the balance of magnetic exchange interactions required for this mechanism is easily disrupted by substrate‐induced strain, lattice disorder, and charge transfer effects.

Consequently, the first experimental example of intrinsic type‐II multiferroicity in the 2D limit was only recently observed in monolayer ${\mathrm{NiI}}_{2}$, where the state is stabilized by strong iodine‐mediated SOC [22, 23, 24]. Validating whether this magnetoelectric coupling is a tunable feature of the TMDH family–rather than a unique property of the heavy iodide–requires moving beyond this single material example. It is therefore essential to investigate isostructural systems where the relevant energy scales are chemically modified. Monolayer ${\mathrm{NiBr}}_{2}$ is the natural candidate: bulk ${\mathrm{NiBr}}_{2}$ is an established type‐II multiferroic, hosting a long‐wavelength cycloidal spin spiral [25, 26] with a spin‐induced, field‐reversible electric polarization below $T_{\mathrm{IC}}\approx 23$ K [27]. In the monolayer limit, theory predicts that the non‐collinear order and the associated multiferroicity may persist [7, 17, 18, 28], and experiments on metal‐supported films have revealed signatures of non‐collinear magnetism [29]; direct experimental evidence of multiferroic order in monolayer ${\mathrm{NiBr}}_{2}$ has, however, remained absent.

Here, we provide direct experimental evidence that multiferroicity is indeed a tunable feature of the nickel dihalide family. Using scanning tunneling microscopy (STM) and spectroscopy (STS), we visualize the ferroelectric domains in monolayer ${\mathrm{NiBr}}_{2}$ as periodic modulations of the local density of states. We confirm the intrinsic nature of the magnetoelectric coupling through reciprocal control experiments: demonstrating the manipulation of magnetic domains via electric fields and the suppression of electric polarization by external magnetic fields. Furthermore, we map the stability limits of the phase, revealing that the spin‐spiral order in ${\mathrm{NiBr}}_{2}$ is energetically less robust than in its iodide counterpart, consistent with altered superexchange interactions and the weaker SOC.

## 2 Results

### 2.1 ${\mathrm{NiBr}}_{2}$ Growth and Observation of Multiferroicity

We grow monolayer ${\mathrm{NiBr}}_{2}$ islands on HOPG (highly oriented pyrolytic graphite) by a single‐source thermal deposition of ${\mathrm{NiBr}}_{2}$ (detailed in the Methods section). By varying the growth parameters, we are able to observe large islands exceeding laterally 200nm as seen in Figure 1a, as well as smaller triangular‐shaped islands, which preferentially grow at the substrate step‐edges (shown in Supporting Information).

**FIGURE 1:**
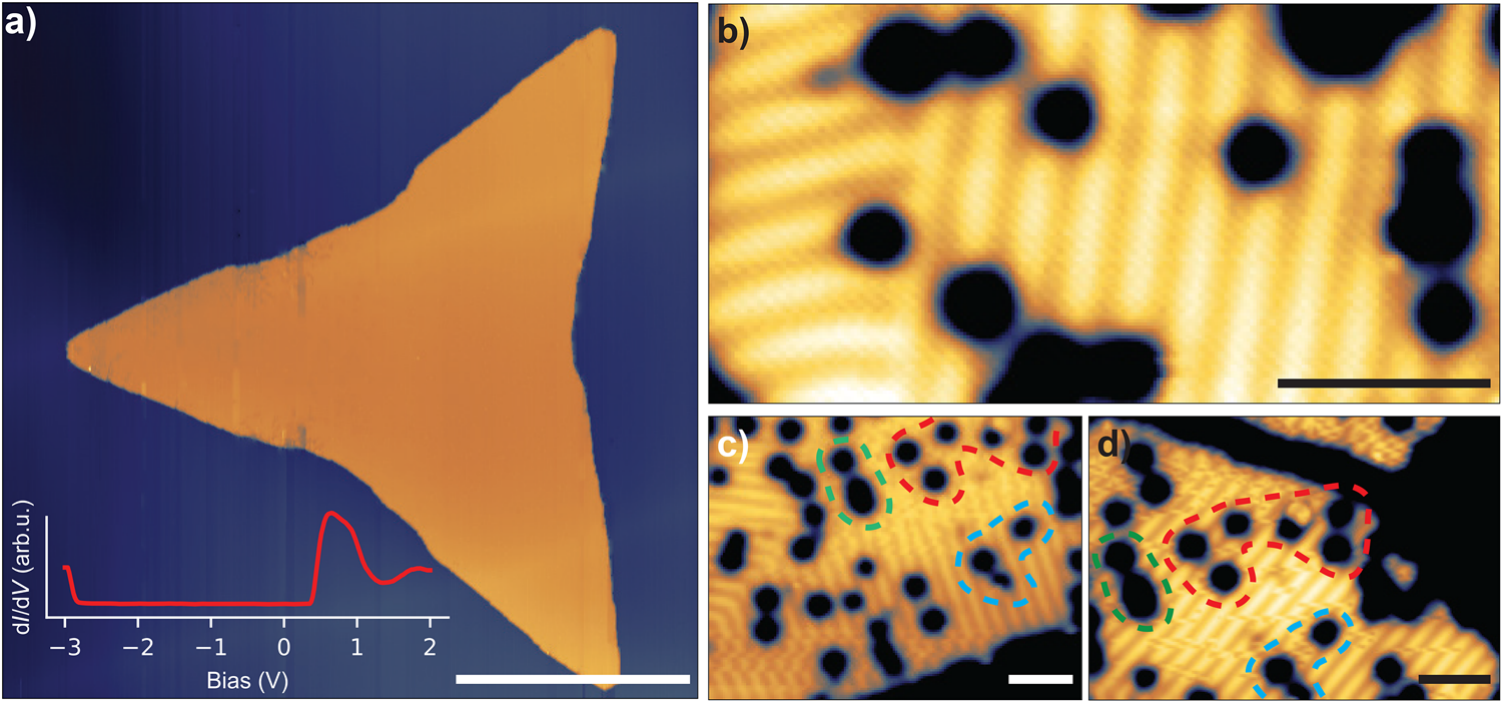
Characterization of monolayer ${\mathrm{NiBr}}_{2}$. (a) Overview topography of monolayer ${\mathrm{NiBr}}_{2}$ island grown on HOPG (2V, 30pA, scale bar 100nm). The inset shows a representative dI/dV spectrum taken on a monolayer ${\mathrm{NiBr}}_{2}$ island. We attribute the small peak at −2.8V to the onset of valence band, although confirmation is difficult due to junction instabilities in this voltage range. (b) Zoom‐in image of monolayer ${\mathrm{NiBr}}_{2}$ taken at 0.3K showing the appearance of stripe‐like pattern when scanning within conduction band (740mV, 20pA, scale bar 10nm). (c,d) Electric manipulation of the domains; a ${\mathrm{NiBr}}_{2}$ island before bias sweep (740mV, 20pA, scale bar 20nm) and after bias sweep (740mV, 10pA, scale bar 20nm). The circled polarons highlight that the scans have been taken at the same area.

The STS measurements locate the onset of the conduction band (CB) of monolayer ${\mathrm{NiBr}}_{2}$ on HOPG at around ∼0.35V (inset of Figure 1a), slightly lower than the onset of the CB of ${\mathrm{NiBr}}_{2}$ grown on Au(111) (∼0.8V) [29]. The STS measurements indicate that the valence band (VB) is located around −2.8V, though we were unable to precisely determine the onset due to junction instabilities at large negative voltages. However, theoretical predictions [19] suggest that the bandgap of the monolayer ${\mathrm{NiBr}}_{2}$ is approximately 4eV, which is consistent with our observation.

Having established the island growth, we proceed to demonstrate the multiferroic nature of monolayer ${\mathrm{NiBr}}_{2}$. The multiferroicity in monolayer ${\mathrm{NiBr}}_{2}$ originates from the same mechanism as in monolayer ${\mathrm{NiI}}_{2}$, which has been investigated both theoretically [6, 7, 17, 18, 28] and experimentally [23, 24, 30, 31]. In monolayer ${\mathrm{NiBr}}_{2}$, Ni atoms adopt a 2+ oxidation state and interact via ferromagnetic nearest‐neighbor ($J_{1}$) and antiferromagnetic third‐nearest‐neighbor ($J_{3}$) exchange coupling. When the ratio $J_{3}/J_{1}$ exceeds a critical value, the competition between exchange interactions favors non‐collinear magnetic ordering in the form of a spin‐spiral characterized by a propagation vector q, whose magnitude depends on $J_{3}/J_{1}$ and is given in units of the reciprocal lattice vectors of the structural unit cell with lattice constant a.

This spin‐spiral ground state, combined with SOC, induces a net electric polarization via the inverse Dzyaloshinskii–Moriya interaction [10], making monolayer ${\mathrm{NiBr}}_{2}$ a type‐II multiferroic, where ferroelectricity emerges as a direct consequence of magnetic order. From the relation between magnetization and emergent electric polarization [6, 11, 12, 24], it follows that electric polarization is modulated in real space with a periodicity equal to half that of the spin spiral. This, in turn, leads to a modulation of local density of states (LDOS) and the electrostatic potential, which can be detected by STM as a stripe‐like contrast with periodicity λ/2, where λ is the magnetic modulation wavelength. STM thus provides a direct means to visualize the multiferroic order without the need for spin‐polarized techniques.

Figure 1b shows the characteristic stripe‐like pattern, previously observed for monolayer ${\mathrm{NiI}}_{2}$ on HOPG [24], that appears when scanning within the conduction band. As shown in Figure 1b, we regularly observe domain formation with stripes rotated by 60°, corresponding to the threefold symmetry of ${\mathrm{NiBr}}_{2}$. We identify the charged defects (dark areas in the topography images) as polarons ‐ charge carriers trapped in a lattice distortion that locally bend the conduction band onset upward (detailed in the Supporting Information) [32, 33]. Under certain scanning conditions, they can be manipulated across the island, but we have found no evidence of their interplay with the multiferroic order.

As mentioned above, the defining property of type‐II multiferroics is the inherent coupling of ferroelectric and magnetic orders, indicating that the magnetic spin‐spiral domains are tunable by external electric fields. To demonstrate this coupling in ${\mathrm{NiBr}}_{2}$, we performed a bias voltage sweep from 1V to 10V in feedback mode (setpoint 200pA) on the HOPG substrate adjacent to the ${\mathrm{NiBr}}_{2}$ island to mitigate the creation of polarons on the monolayer – a phenomenon also reported for monolayer ${\mathrm{CoCl}}_{2}$ [32, 33]. Figure 1c,d presents the ${\mathrm{NiBr}}_{2}$ monolayer before and after the bias sweep, respectively. The orientation of the multiferroic domains changes distinctly, indicating that the spin‐spiral order has been reoriented. This sensitivity of the magnetic structure to electric effects confirms the coupling between electric and magnetic orders, establishing monolayer ${\mathrm{NiBr}}_{2}$ as a type‐II multiferroic.

### 2.2 Atomic‐Scale Characterization

To probe the multiferroic ordering at the atomic limit, we imaged the ${\mathrm{NiBr}}_{2}$ layer at bias voltages corresponding to the conduction band (750mV, Figure 2a) and within the bandgap (250mV, Figure 2b). Remarkably, the stripe pattern persists in both energy ranges. Figure 2b, taken within the bandgap, simultaneously resolves the upper bromine atomic lattice and the ferroelectric modulation. This differs from the monolayer ${\mathrm{NiI}}_{2}$, where the multiferroic contrast is only visible when scanning within the conduction band [24].

**FIGURE 2:**
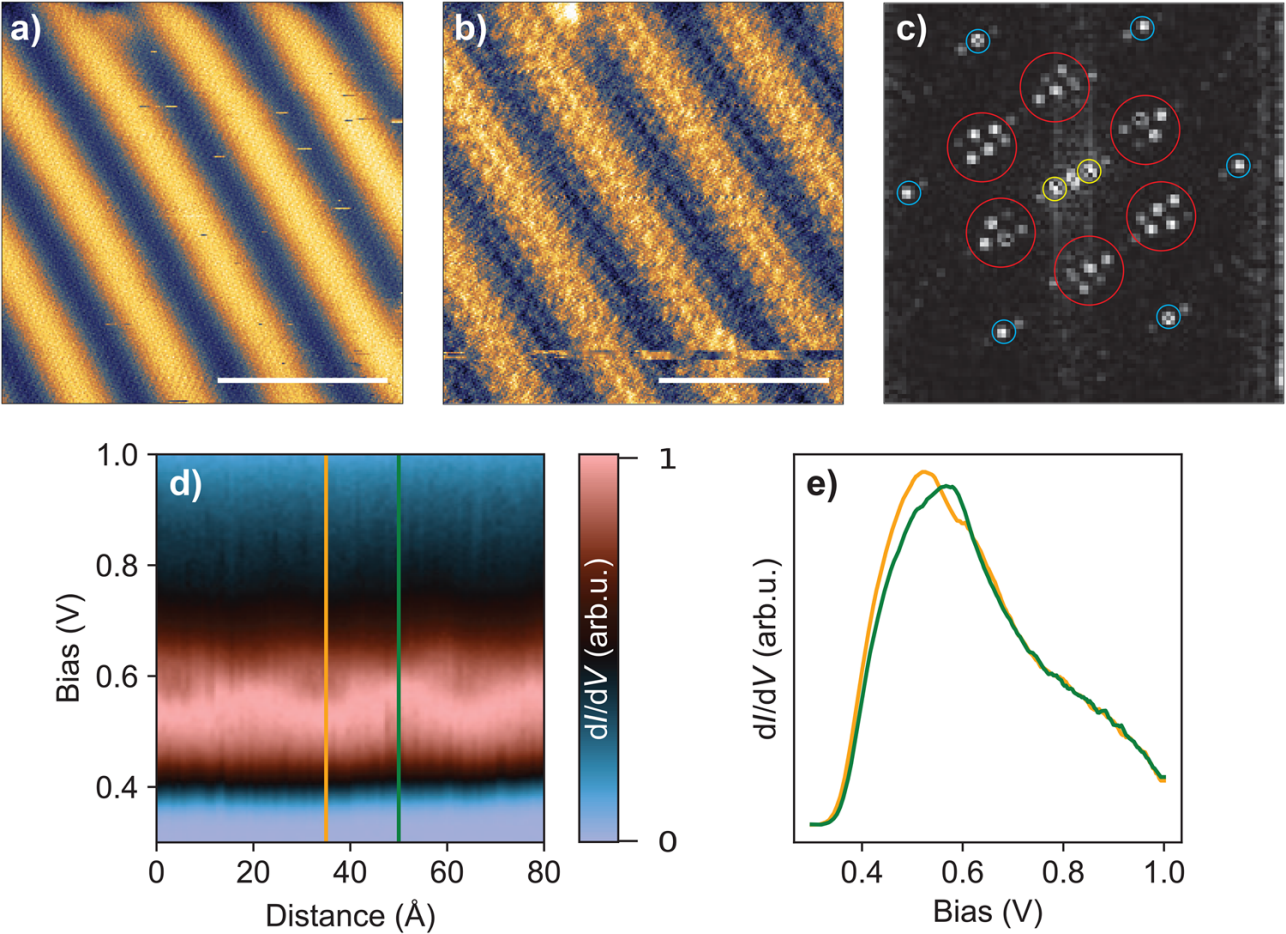
Atomic‐scale imaging and spectroscopy of the multiferroic stripes. (a) High‐resolution topography of multiferroic stripes on monolayer ${\mathrm{NiBr}}_{2}$ acquired within the conduction band (750mV, 10pA; scale bar: 5nm). (b) Topography of the same area taken within the bandgap, where both the atomic lattice and multiferroic stripes are simultaneously resolved (250mV, 10pA; scale bar: 5nm). (c) FFT of image (b). Blue circles mark the reciprocal lattice points of the ${\mathrm{NiBr}}_{2}$ unit cell, yellow circles indicate the multiferroic modulation, and red circles correspond to the moiré pattern arising from the lattice mismatch between HOPG and ${\mathrm{NiBr}}_{2}$. (d) Spectroscopic line profile of the conduction band (CB) acquired across the multiferroic stripes. A modulation is visible between 0.5V and 0.6V. (e) Individual point spectra taken at the positions indicated by vertical lines in d. A modulation of the LDOS, a shift of the CB maximum, and contrast inversions are observed between 0.4V and 0.6V.

While complete understanding of multiferroic contrast inside the band gap requires theoretical work beyond the scope of this study, we note two physically motivated mechanisms that likely contribute jointly. First, at sub‐gap energies the tunneling current is dominated by tunneling into substrate‐derived states, since the 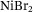 monolayer has no intrinsic states at that energy. The ${\mathrm{NiBr}}_{2}$ overlayer acts as a structured tunneling filter, and the ferroelectric polarization periodically modulates the height of this barrier resulting in a stripe modulation. Second, the proximity of the ${\mathrm{NiBr}}_{2}$ modulates the density of states (DOS) of the underlying HOPG, which then inherits the periodic modulation imposed by the multiferroic order. A quantitative deconvolution of their contributions is an interesting direction for future theoretical work.

From the atomic‐resolution topography and its corresponding Fast Fourier Transform (FFT) (Figure 2c), we determine the experimental lattice constant of the structural unit cell to be a=3.6Å and the stripe periodicity to be $L_{s}=28\;$Å. As the observed stripe pattern reflects the modulation of the electric polarization, its periodicity corresponds to half the spin spiral wavelength. This implies a full spin spiral periodicity of 56Å, or approximately 15.6 times the lattice constant (λ=15.6a), while the propagation vector q deviates by approximately 7° from the $\bar{\Gamma K}$ direction. Notably, we find that the stripe periodicity in ${\mathrm{NiBr}}_{2}$ is significantly larger than that of ${\mathrm{NiI}}_{2}$. This increase in λ (decrease of q) marks a shift in the ratio of competing exchange constants, placing ${\mathrm{NiBr}}_{2}$ closer to the transition boundary between the non‐collinear spiral and a collinear ferromagnetic state [7]. The additional spots near the Γ point in the FFT (Figure 2c) are attributed to a moiré pattern arising from the lattice mismatch between the ${\mathrm{NiBr}}_{2}$ monolayer and the HOPG substrate.

Next, we investigate the LDOS variations associated with the spatial electric modulation. Figure 2d displays STS measurements taken along a line perpendicular to the stripe direction. A periodic modulation of CB is detected, which can be investigated in more detail when individual slices of line spectra are taken at the extrema of the stripe modulation (Figure 2e). This reveals the energetic position of the CB maxima to shift by approximately 37mV (extraction shown in Supporting Information). We attribute this LDOS modification to the underlying ferroelectric order, which induces a periodic electrostatic potential that reshapes the local band structure.

### 2.3 Phase Stability and Robustness

To assess the stability of the multiferroic state and map the phase transition into the ferromagnetic phase, we examined the dependence of the spin‐spiral order on temperature and external magnetic fields. The non‐collinear magnetic ground state in ${\mathrm{NiBr}}_{2}$ relies on a delicate balance of exchange interactions and SOC; consequently, thermal fluctuations or the Zeeman interaction are expected to destabilize the spiral propagation [6, 7, 13, 34, 35]. Figure 3a confirms the presence of multiferroic domains at a temperature of 3.5K. As the temperature is increased, the stripe contrast progressively decreases, disappearing completely at $T_{C}∼5.5\;K$ (Figure 3b and Figure S3), marking the phase transition from the helimagnetic spin‐spiral state to the paramagnetic phase. We subsequently applied an out‐of‐plane magnetic field to probe the magnetic susceptibility of these domains. While the stripe pattern is unaffected at low fields (Figure 3c), the contrast fades with increasing field strength until the stripes disappear entirely at $B_{C}∼4\;T$ (Figure 3d and Figures S4 and S5), indicating that the external field has overcome the frustration of the exchange interactions to force a field‐polarized ferromagnetic alignment.

**FIGURE 3:**
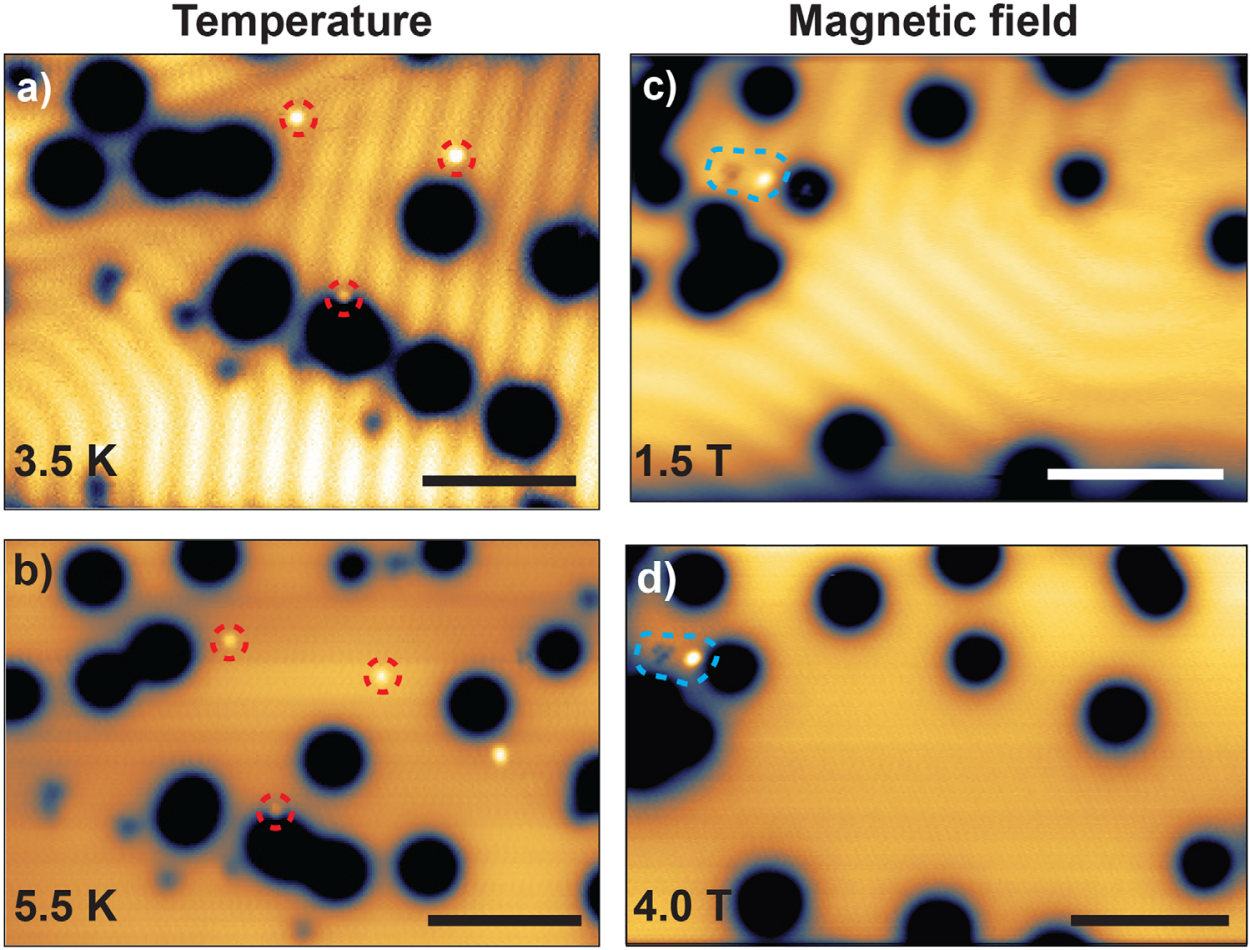
Manipulation of multiferroicity through temperature and magnetic field. (a,b) Temperature dependence measurement; Scans taken at 3.5 K (740mV, 20pA, scale bar 10nm) and at 5.5 K (740mV, 20pA, scale bar 10nm). (c,d) Magnetic field dependence; Scans taken at 1.5 T (760mV, 30pA, scale bar 10nm) and at 4.0 T (760mV, 30pA, scale bar 10nm). The red (a,b) and blue (c,d) circles show stationary defects, showing that the scans have been taken at same areas.

The observed critical values ($T_{C}∼5.5\;K$, $B_{C}∼4\;T$) represent a significant reduction in stability compared to monolayer ${\mathrm{NiI}}_{2}$, where the multiferroic phase persists up to 15K and remains robust in fields exceeding 11T [35]. This comparative fragility in ${\mathrm{NiBr}}_{2}$ is attributed to the concerted effect of modified superexchange interactions and the weaker SOC of the lighter bromine ligand [6, 7, 17, 21]. The former sets a lower overall magnetic energy scale bringing the system closer to the ferromagnetic transition boundary (as exemplified by the larger spin spiral periodicity λ), while the latter reduces the anisotropy barrier protecting the non‐collinear order against both thermal fluctuations and field‐induced alignment. Consequently, whereas high fields in ${\mathrm{NiI}}_{2}$ only reorient the spiral into a single domain, the less robust magnetic order in ${\mathrm{NiBr}}_{2}$ allows for a complete suppression of the spin‐spiral order at relatively low field strengths.

Finally, these measurements provide further evidence of the magnetoelectric coupling established in the previous sections. Since our STM tip is non‐spin‐polarized, the contrast in our images arises solely from the electric order – specifically, the modulation of the local electrostatic potential [35]. The fact that an external magnetic field, which couples directly to the atomic spins, suppresses this electric contrast confirms that the electric order is directly linked to the underlying magnetic structure. Together with the electric‐field manipulation of the domains shown in Figure 1, this confirms the mutual coupling of the order parameters characteristic of type‐II multiferroicity.

## 3 Conclusions

In conclusion, we have established the type‐II multiferroic nature of monolayer ${\mathrm{NiBr}}_{2}$ on HOPG. STM imaging resolves the ferroelectric domains arising from the non‐collinear spin‐spiral order, while spectroscopy confirms that this order induces a periodic electrostatic potential modulation that locally alters the LDOS. We demonstrated inherent magnetoelectric coupling through reciprocal manipulation, successfully reorienting magnetic domains via electric fields and suppressing the ferroelectric order with external magnetic fields.

Comparing ${\mathrm{NiBr}}_{2}$ with isostructural ${\mathrm{NiI}}_{2}$ (Table 1) shows how the two ingredients of type‐II multiferroicity respond to halide substitution. The longer spin‐spiral periodicity in ${\mathrm{NiBr}}_{2}$, and its proximity to the collinear‐ferromagnetic boundary, follow from the smaller $J_{3}/J_{1}$ frustration ratio, an effect set by the isotropic exchange and essentially independent of spin‐orbit coupling. The weaker spin–orbit coupling of the bromine ligand enters separately, and is expected to reduce both the magnetic anisotropy that stabilizes the non‐collinear order, which is consistent with the lower critical temperature and field, and the inverse‐Dzyaloshinskii–Moriya polarization that produces the multiferroic contrast. That these distinct exchange‐ and SOC‐controlled properties all shift in the same direction under halide substitution demonstrates that the multiferroic mechanism established for ${\mathrm{NiI}}_{2}$ extends to ${\mathrm{NiBr}}_{2}$ and is tunable via the ligand alteration. This establishes the nickel dihalides as a model platform for engineering magnetoelectric phases at the atomic limit.

**TABLE 1:** Comparison of the key parameters of monolayer multiferroic ${\mathrm{NiI}}_{2}$ and ${\mathrm{NiBr}}_{2}$ (${\mathrm{NiBr}}_{2}$ from this work; ${\mathrm{NiI}}_{2}$ from cited references). The two reported values for $T_{C}$ of ${\mathrm{NiI}}_{2}$ differ depending on the measurement technique and the underlying substrate (15K [35] for ${\mathrm{NiI}}_{2}$/HOPG with STM and 21K [23] for ${\mathrm{NiI}}_{2}$ on hBN with second‐harmonic generation). The stripe periodicity is half the spin‐spiral wavelength; $T_{C}$ and $B_{C}$ are the temperature and field above which the stripe contrast is fully suppressed.

	${\mathrm{NiI}}_{2}$	${\mathrm{NiBr}}_{2}$
Measured stripe periodicity	18Å [24]	28Å
Inferred spin‐spiral periodicity	36Å [24]	56Å
Transition temperature ($T_{C}$)	15K [35]/21K [23]	5.5K
Critical magnetic field ($B_{C}$)	≫ 11T [35]	4T
$J_{3}$/$J_{1}$ exchange interaction	**1.5** [7]	**1.25** [7]

## 4 Methods

### 4.1 Sample Preparation

The ${\mathrm{NiBr}}_{2}$ samples were grown in an ultra‐high vacuum (UHV) molecular beam epitaxy (MBE) system on HOPG substrates. The HOPG was exfoliated under ambient conditions and subsequently transferred to the preparation chamber with a base pressure of 2.0 × 10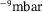, where it was outgassed at 250°C. ${\mathrm{NiBr}}_{2}$ was deposited from a single‐source anhydrous powder using a Knudsen cell.

The substrate temperature was ∼175°C for all samples, with the ${\mathrm{NiBr}}_{2}$ evaporation temperature ∼415°C. Growth time varied between 45–50 min. Post‐annealing was done for 5–15 min, around 205°C–215°C. Our experience indicates that the substrate and evaporation temperatures are most critical for achieving the optimal growth.

### 4.2 STM Measurements

The experiments were carried out with low temperature (LT) UHV Unisoku USM1300 STM system. Base temperature for measurements was ∼330 mK. All of the measurements were performed with Pt/Ir tips. For the spectroscopy measurements, we use a conventional lock‐in technique at a frequency of 757 Hz with 25 mV modulation.

## Author Contributions

**Aleš Cahlík**: Methodology, Validation, Investigation, Formal analysis, Writing – Original Draft, Supervision, Funding acquisition. **Antti Karjasilta**: Conceptualization, Methodology, Validation, Investigation, Formal analysis, Writing – Original Draft. **Anshika Mishra**: Validation, Investigation. **Robert Drost**: Formal analysis, Visualization. **Mohammad Amini**: Conceptualization, Investigation. **Javaria Arshad**: Investigation. **Büşra Gamze Arslan**: Investigation. **Peter Liljeroth**: Conceptualization, Supervision, Funding acquisition, Writing – Review and Editing.

## Conflicts of Interest

The authors declare no conflicts of interest.

## Supporting information


**Supporting File**: advs77081‐sup‐0001‐SuppMat.pdf.

## Data Availability

All data presented in the main text or in the Supporting Information are available on the Zenodo repository: https://doi.org/10.5281/zenodo.20508274.

## References

[1] P. Ajayan , P. Kim , and K. Banerjee , “Two‐Dimensional van der Waals Materials,” Physics Today 69, no. 9 (September 2016): 38–44, 10.1063/PT.3.3297.

[2] K. S. Burch , D. Mandrus , and J.‐G. Park , “Magnetism in Two‐Dimensional van der Waals Materials,” Nature 563, no. 7729 (November 2018): 47–52, 10.1038/s41586-018-0631-z. 30382199

[3] , H. Li , S. Ruan , and Y. Zeng , “Intrinsic van der Waals Magnetic Materials from Bulk to the 2D Limit: New Frontiers of Spintronics,” Advanced Materials 31, no. 27 (May 2019): 1900065, 10.1002/adma.201900065. 31069896

[4] M. Wang , C. Huang , C. Cheung et al., “Prospects and Opportunities of 2D van der Waals Magnetic Systems,” Annalen der Physik 532, no. 5 (March 2020): 1900452, 10.1002/andp.201900452.

[5] M. Haavisto , J. L. Lado , and A. Otero Fumega , “Topological Multiferroic Order in Twisted Transition Metal Dichalcogenide Bilayers,” SciPost Physics 13, no. 3 (September 2022): 052, 10.21468/SciPostPhys.13.3.052.

[6] A. O. Fumega and J. L. Lado , “Microscopic Origin of Multiferroic Order in Monolayer ${\mathrm{NiI}}_{2}$ ,” 2D Materials 9, no. 2 (February 2022): 025010, 10.1088/2053-1583/ac4e9d.

[7] K. Riedl , D. Amoroso , S. Backes et al., “Microscopic Origin of Magnetism in Monolayer 3d Transition Metal Dihalides,” Physical Review B 106, no. 3 (July 2022): 035156, 10.1103/PhysRevB.106.035156.

[8] L. Xu , Z. Wang , and C. Gao , “Scanning Tunneling Microscope Studies on Low‐Dimensional Transition‐Metal Halides,” Japanese Journal of Applied Physics 63, no. 5 (May 2024): 050805, 10.35848/1347-4065/ad3b05.

[9] J. Deng , D. Guo , Y. Wen et al., “Evidence of Ferroelectricity in an Antiferromagnetic Vanadium Trichloride Monolayer,” Science Advances 11, no. 10 (March 2025): eado6538, 10.1126/sciadv.ado6538. PMC11881914 40043120

[10] H. Katsura , N. Nagaosa , and A. V. Balatsky , “Spin Current and Magnetoelectric Effect in Noncollinear Magnets,” Physical Review Letters 95 (Jul 2005): 057205, 10.1103/PhysRevLett.95.057205. 16090916

[11] M. Mostovoy , “Ferroelectricity in Spiral Magnets,” Physical Review Letters 96 (Feb 2006): 067601, 10.1103/PhysRevLett.96.067601. 16606047

[12] J. Hu , “Microscopic Origin of Magnetoelectric Coupling in Noncollinear Multiferroics,” Physical Review Letters 100 (Feb 2008): 077202, 10.1103/PhysRevLett.100.077202. 18352590

[13] Y. Tokura and S. Seki , “Multiferroics with Spiral Spin Orders,” Advanced Materials 22, no. 14 (2010): 1554–1565, 10.1002/adma.200901961. 20496385

[14] D. Pantel , S. Goetze , D. Hesse , and M. Alexe , “Reversible Electrical Switching of Spin Polarization in Multiferroic Tunnel Junctions,” Nature Materials 11, no. 4 (April 2012): 289–293, 10.1038/nmat3254. 22367005

[15] T. Hu and E. Kan , “Progress and Prospects in Low‐Dimensional Multiferroic Materials,” WIREs Computational Molecular Science 9, no. 5 (November 2019): e1409, 10.1002/wcms.1409.

[16] Y. Gao , M. Gao , and Y. Lu , “Two‐Dimensional Multiferroics,” Nanoscale 13, no. 46 (November 2021): 19324–19340, 10.1039/D1NR06598J. 34816857

[17] H.‐S. Yu , X.‐S. Ni , D.‐X. Yao , and K. Cao , “Microscopic Origin of Magnetoferroelectricity in Monolayer ${\mathrm{NiBr}}_{2}$ and ${\mathrm{NiI}}_{2}$ ,” Physical Review B 111, no. 9 (March 2025): 094440, 10.1103/PhysRevB.111.094440.

[18] J. Sødequist and T. Olsen , “Type II Multiferroic Order in Two‐Dimensional Transition Metal Halides from First Principles Spin‐Spiral Calculations,” 2D Materials 10, no. 3 (May 2023): 035016, 10.1088/2053-1583/acd4d0.

[19] M. Lu , Q. Yao , C. Xiao , C. Huang , and E. Kan , “Mechanical, Electronic, and Magnetic Properties of ${\mathrm{NiX}}_{2}$ (X = Cl, Br, I) Layers,” ACS Omega 4, no. 3 (March 2019): 5714–5721, 10.1021/acsomega.9b00056. PMC6648776 31459724

[20] X.‐F. Luo , X. He , R. Wang , H. Xiang , and J.‐Z. Zhao , “Orbital Order Triggered Out‐of‐Plane Ferroelectricity in Magnetic Transition Metal Dihalide Monolayers,” Nano Letters 25, no. 25 (June 2025): 10145–10151, 10.1021/acs.nanolett.5c02124. 40492644

[21] X. Li , C. Xu , B. Liu , X. Li , L. Bellaiche , and H. Xiang , “Realistic Spin Model for Multiferroic ${\mathrm{NiI}}_{2}$ ,” Physical Review Letters 131, no. 3 (July 2023): 036701, 10.1103/PhysRevLett.131.036701. 37540870

[22] H. Ju , Y. Lee , K.‐T. Kim et al., “Possible Persistence of Multiferroic Order Down to Bilayer Limit of van der Waals Material ${\mathrm{NiI}}_{2}$ ,” Nano Letters 21, no. 12 (June 2021): 5126–5132, 10.1021/acs.nanolett.1c01095. 34096728

[23] Q. Song , C. A. Occhialini , E. Ergeşen et al., “Evidence for a Single‐Layer van der Waals Multiferroic,” Nature 602, no. 7898 (February 2022): 601–605, 10.1038/s41586-021-04337-x. 35197619

[24] M. Amini , A. O. Fumega , H. González‐Herrero et al., “Atomic‐Scale Visualization of Multiferroicity in Monolayer ${\mathrm{NiI}}_{2}$ ,” Advanced Materials 36, no. 18 (January 2024): 2311342, 10.1002/adma.202311342. 38241258

[25] A. Adam , D. Billerey , C. Terrier et al., “Neutron Diffraction Study of the Commensurate and Incommensurate Magnetic Structures of ${\mathrm{NiBr}}_{2}$ ,” Solid State Communications 35 (1980): 1–5, 10.1016/0038-1098(80)90757-7.

[26] S. Babu , K. Prokeš , Y. K. Huang , F. Radu , and S. K. Mishra , “Magnetic‐Field‐Induced Incommensurate to Collinear Spin Order Transition in ${\mathrm{NiBr}}_{2}$ ,” Journal of Applied Physics 125 (2019): 093902, 10.1063/1.5066625.

[27] Y. Tokunaga , D. Okuyama , T. Kurumaji et al., “Multiferroicity in ${\mathrm{NiBr}}_{2}$ with Long‐Wavelength Cycloidal Spin Structure on a Triangular Lattice,” Physical Review B 84, no. 6 (August 2011): 060406, 10.1103/PhysRevB.84.060406.

[28] T. B. Prayitno , E. Budi , and Y. P. Sarwono , “Influence of Doping and Electric Field on Magnetism of ${\mathrm{NiBr}}_{2}$ Monolayer: Possible Potentiality for Multiferroicity and Thermoelectricity,” Journal of Physics and Chemistry of Solids 188 (May 2024): 111910., 10.1103/PhysRevB.84.060406.

[29] D. Bikaljević , C. González‐Orellana , M. Peña‐Díaz et al., “Noncollinear Magnetic Order in Two‐Dimensional ${\mathrm{NiBr}}_{2}$ Films Grown on Au(111),” ACS Nano 15, no. 9 (September 2021): 14985–14995, 10.1021/acsnano.1c05221. PMC8482757 34491033

[30] M.‐P. Miao , N. Liu , W.‐H. Zhang et al., “Spin‐Resolved Imaging of Atomic‐Scale Helimagnetism in Mono‐ and Bilayer ${\mathrm{NiI}}_{2}$ ,” Proceedings of the National Academy of Sciences of the United States of America 122, no. 39 (2025): e2422868122, 10.1073/pnas.2422868122. PMC12501136 40986345

[31] H. Wang , T. Jiang , W. Pan et al., “Microscopic Evidence of Spin‐Driven Multiferroicity and Topological Spin Textures in Monolayer ${\mathrm{NiI}}_{2}$ ,” Physical Review Letters 136, no. 2 (January 2026): 026402, https://journals.aps.org/prl/abstract/10.1103/4hzc‐bm2f. 10.1103/4hzc-bm2f 41616348

[32] M. Cai , M.‐P. Miao , Y. Liang et al., “Manipulating Single Excess Electrons in Monolayer Transition Metal Dihalide,” Nature Communications 14, no. 1 (June 2023): 3691, https://www.nature.com/articles/s41467‐023‐39360‐1. 10.1038/s41467-023-39360-1 PMC10284909 37344472

[33] H. Liu , A. Wang , P. Zhang et al., “Atomic‐Scale Manipulation of Single‐Polaron in a Two‐Dimensional Semiconductor,” Nature Communications 14, no. 1 (June 2023): 3690, https://www.nature.com/articles/s41467‐023‐39361‐0. 10.1038/s41467-023-39361-0 PMC10284845 37344475

[34] S. Hayami , S.‐Z. Lin , and C. D. Batista , “Bubble and Skyrmion Crystals in Frustrated Magnets with Easy‐Axis Anisotropy,” Physical Review B 93 (May 2016): 184413, 10.1103/PhysRevB.93.184413.

[35] M. Amini , T. V. C. Antão , L. Jing et al., “Observation of Electromagnons in a Monolayer Multiferroic,” (2025), arXiv:2510.08253, 10.48550/arXiv.2510.08253.

