# Supplementary material for: Probing Type‐II Multiferroicity in Monolayer NiBr2
Source: Adv Sci (Weinh). 2026 Aug 13:e77081. Online ahead of print. doi: 10.1002/advs.77081 (PMC13472127; doi:10.1002/advs.77081)
Supplement: Supplementary file 1 [file ADVS-9999-e77081-s001.pdf]

Supplementary Information:
Probing Type-II Multiferroicity in Monolayer $NiBr_2$

Aleš Cahlík,[1,*] Antti Karjasilta,[1,*] Anshika Mishra,[1] Robert Drost,[1] Mohammad Amini,[1] Javaria Arshad,[1] Büşra Gamze Arslan,[1] and Peter Liljeroth[1]

[1] *Aalto University, Department of Applied Physics, 00076 Aalto, Finland*

A. Growth of monolayer $NiBr_2$ on HOPG

While we successfully achieved the growth of large, isolated monolayer $NiBr_2$ islands (see Figure 1a in the main text), the deposition process is predominantly characterized by step-edge decoration (Fig. S1a-b). Nucleation appears to initiate preferentially along the step edges, leading to the formation of triangular-shaped islands significantly smaller than the large ones found on the open terraces. Furthermore, for the used preparation parameters, we did not observe the formation of a continuous bilayer; instead, excess material tends to form disordered clusters on top of the $NiBr_2$ monolayer (Fig. S1a).

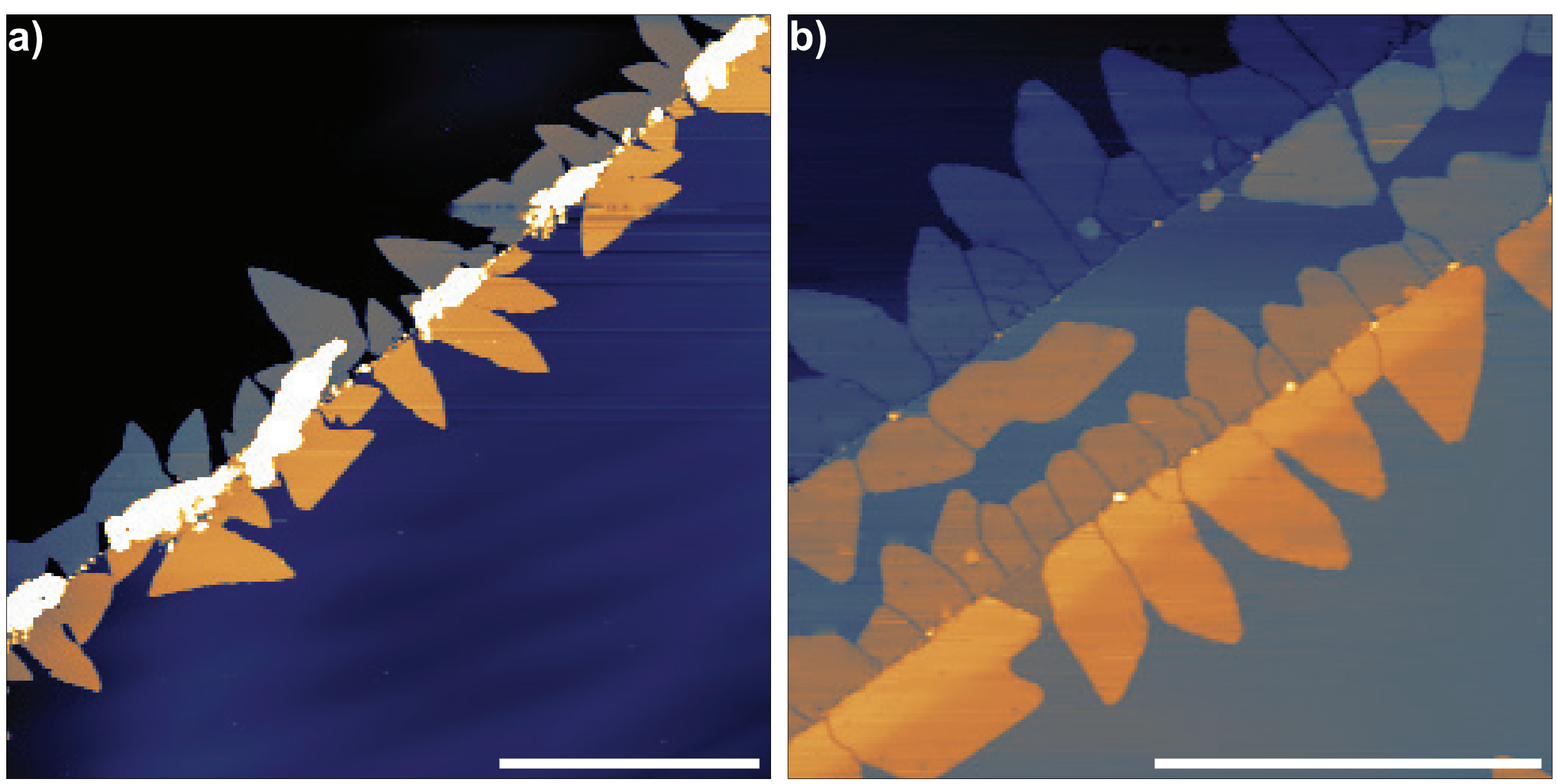

Fig. S1. **Growth morphology of $NiBr_2$ on HOPG. a, b** Large-scale STM topography images of step-edge nucleation. In **a**, the nucleation of excess material on top of the $NiBr_2$ layer is visible near the step edge. Scanning parameters: (**a**) 2 V, 15 pA, scale bar 200 nm; (**b**) 1 V, 20 pA, scale bar 200 nm.

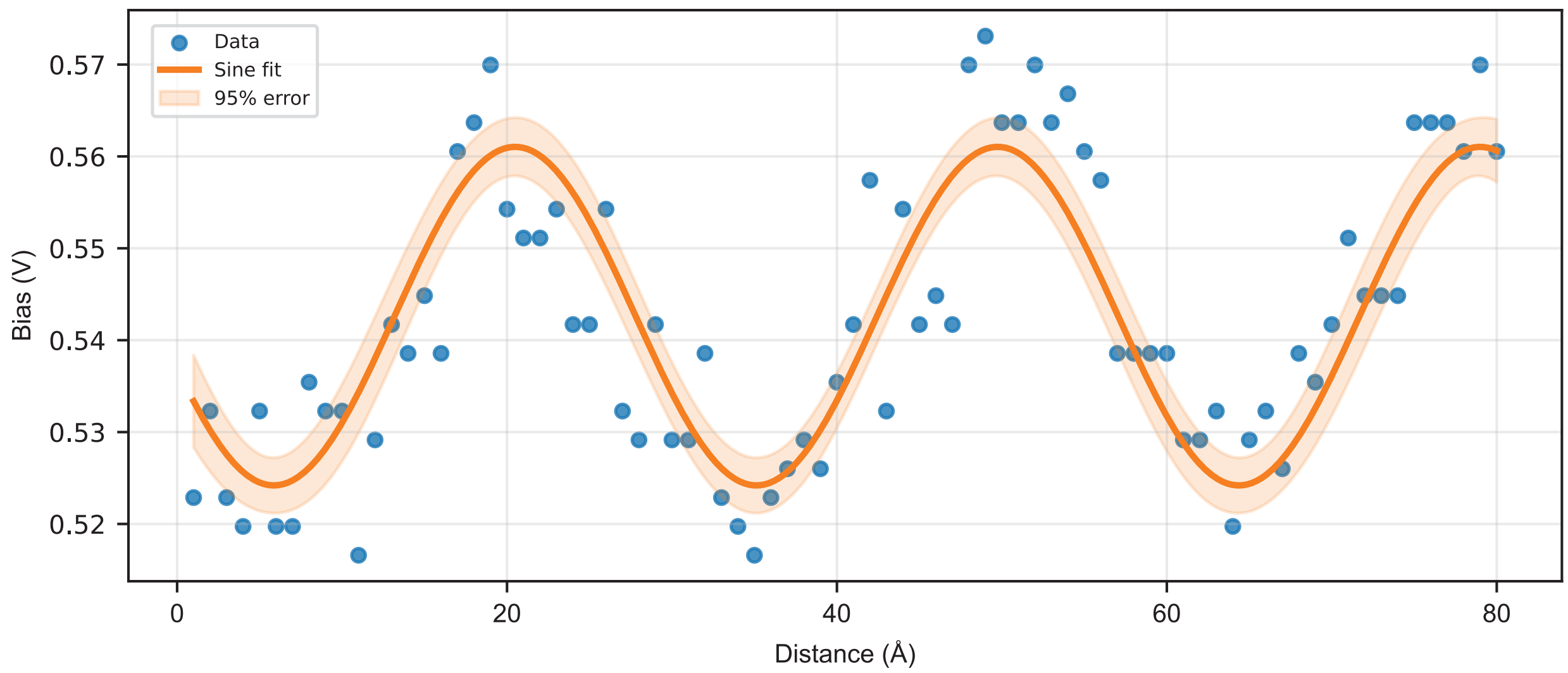

Fig. S2. **Spatial modulation of the CB maximum.** The bias voltage corresponding to the maximum dI/dV intensity of the CB is plotted as a function of distance across the multiferroic stripes. From the overlaid sine fit, we extract an apparent shift of the CB maximum of approximately 37 mV.

B. Analysis of LDOS modulation

To quantify the LDOS modulation, we extracted the CB maximum energy from the line profile plotted in Fig. 2d for each individual spectrum. From the sine fit to this data, we extract an apparent CB maximum shift of approximately 37 mV (Fig. S2).

C. Temperature dependence of the stripes

The multiferroic stripes and domains are clearly resolved at a base temperature of 0.3 K (see Figure 1b in the main text). Upon heating of the sample, the contrast of the stripe gradually decreases, eventually disappearing completely at 5.5 K (Fig. S3). Subsequent cooling restores the visibility of the domains and stripe patterns. These observations indicate that the critical temperature (T_C) for the magnetic phase transition from the spin-spiral to the paramagnetic state in monolayer $NiBr_2$ lies in the range of 5.0–5.5 K, however slight variations were observed between individual islands and domains (on the order of ∼1 K).

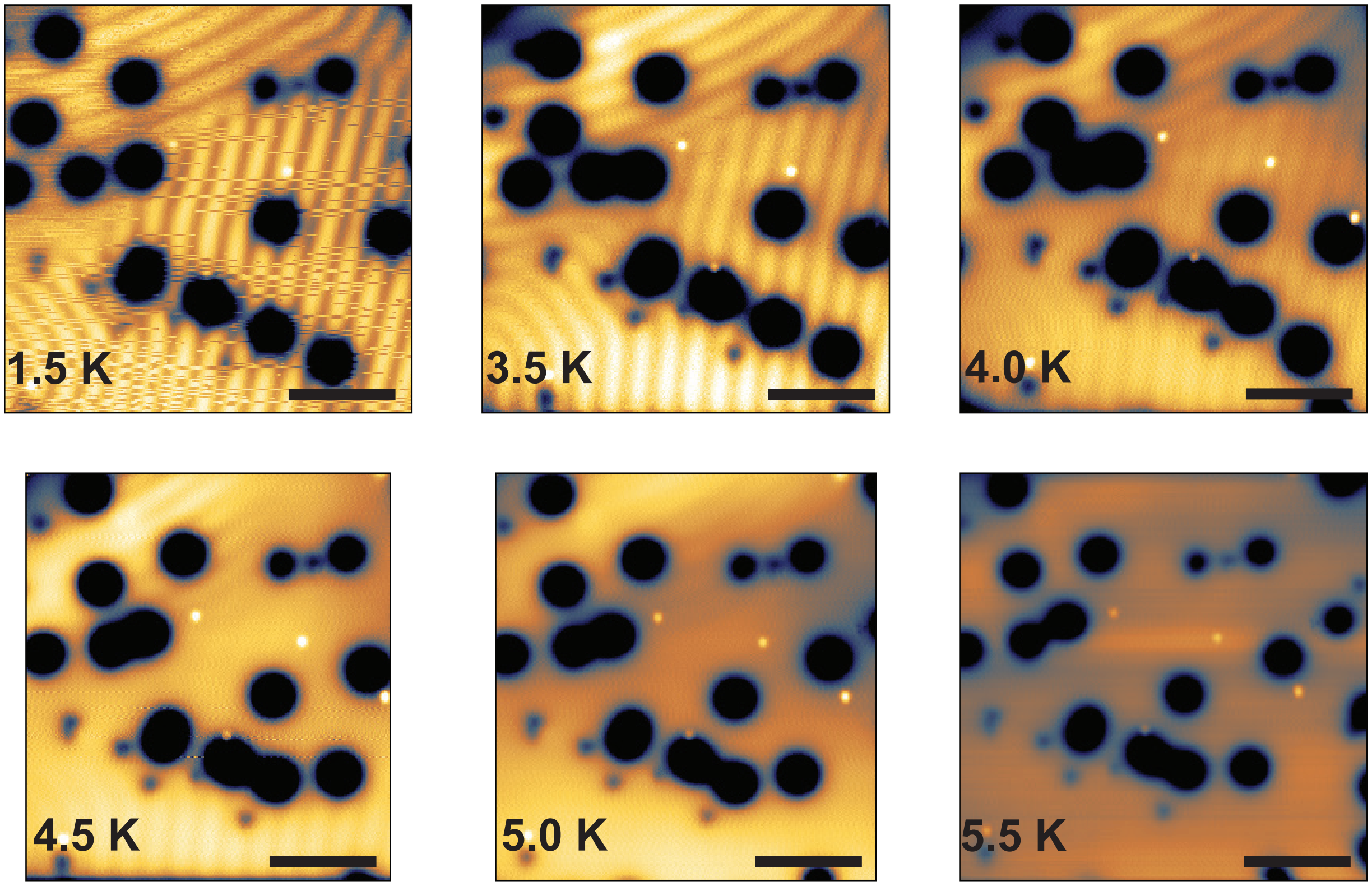

Fig. S3. **Temperature evolution of the multiferroic domains.** Topography images acquired at temperatures ranging from 1.5 K to 5.5 K showing the gradual disappearance of ferroelectric domains. All scans taken at 740 mV, 20 pA, scale bars 10 nm.

D. Magnetic field dependence of the stripes

The evolution of a multiferroic domain under external magnetic fields ranging from 0.5 T to 4.0 T is presented in Fig. S4. The stripe pattern remains largely unaffected up to 3.5 T, at which point the contrast becomes noticeably weaker. At 4.0 T, the stripes disappear completely across all domains, signaling the transition to the ferromagnetic state. Upon decreasing the magnetic field, the stripes reappear, maintaining their original orientation (Fig. S4, first and last panels).

To investigate the effect of the magnetic field on the multiferroic stripes quantitatively, we extracted line profiles across the stripes (location marked in the first panel of Fig. S4) and, after removing linear background, fitted them with a sinusoid to obtain the stripe amplitude as a function of field (Fig. S5). Within the limits set by the signal-to-noise of the profiles and the small number of field values measured, the amplitude decreases with increasing field. At 3.5 T the stripe modulation could barely be resolved, and at 4.0 T no modulation could be extracted from the line profiles.

We note that individual domains are not suppressed at exactly the same field (see Fig. S4, panel 3.5 T), so the complete loss of contrast occurs over a range of roughly 3.5–4.0 T rather than at a single sharp threshold.

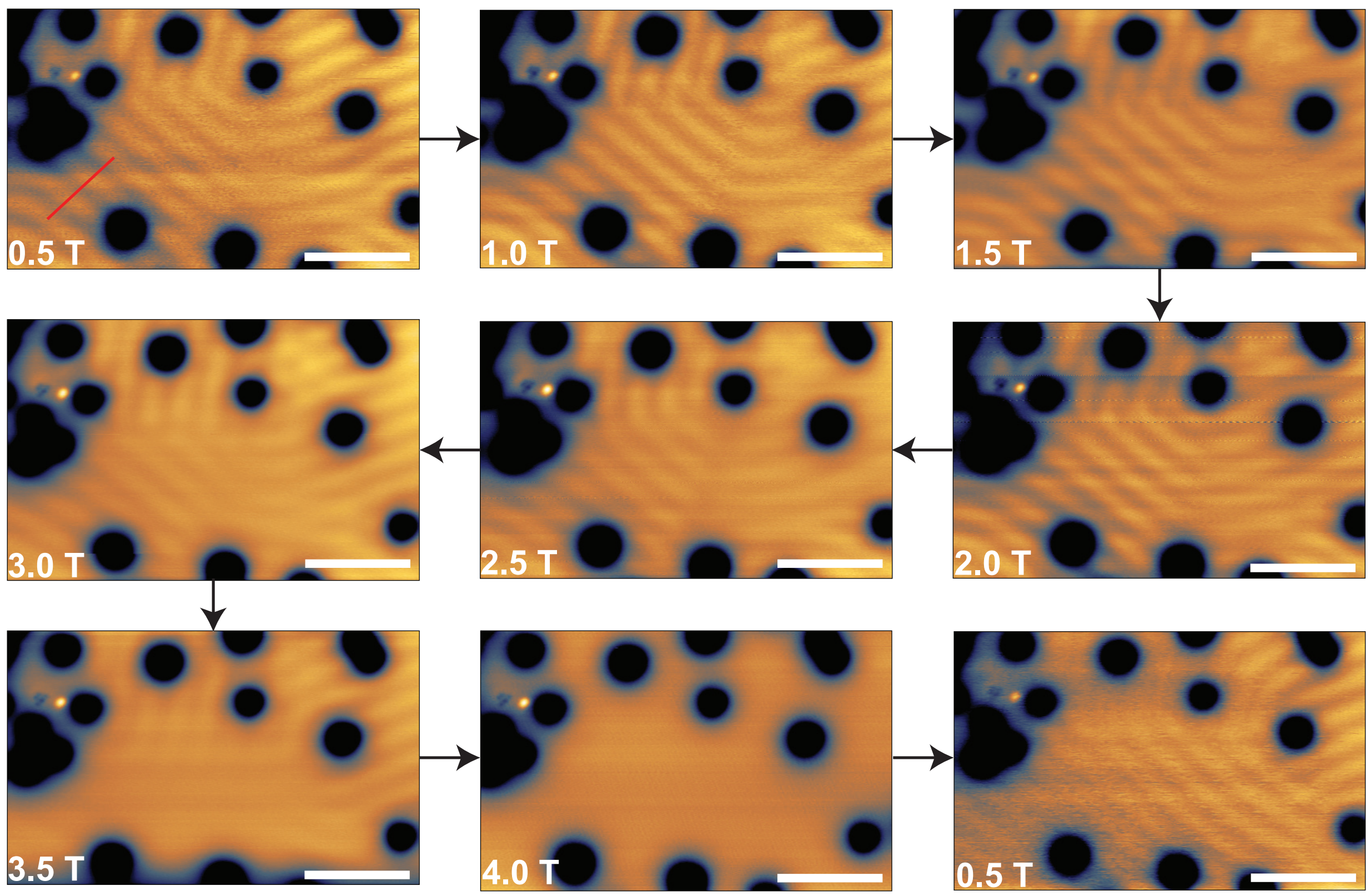

Fig. S4. **Evolution of the multiferroic order in an external magnetic field.** Topography images acquired in out-of-plane magnetic fields ranging from 0.5 T to 4.0 T and back to 0.5 T. All scans taken at 760 mV, 30 pA, scale bars 10 nm.

E. Polaron defects

Depending on the preparation conditions, we observe varying density of point defects on monolayer $NiBr_2$ islands. Some of these defects manifest as dark depressions in topography, as shown in Fig. S6a, that can be manipulated across the island depending on the scanning parameters. For better characterization, we performed scanning tunneling spectroscopy (STS) line profiles across these sites. As shown in Supplementary Figure S6b, the defects induce a distinct upward shift in the CB onset. These findings confirm that the defects are charged, consistent with their identification as polarons—quasiparticles formed by the coupling of charge carriers to lattice distortions.

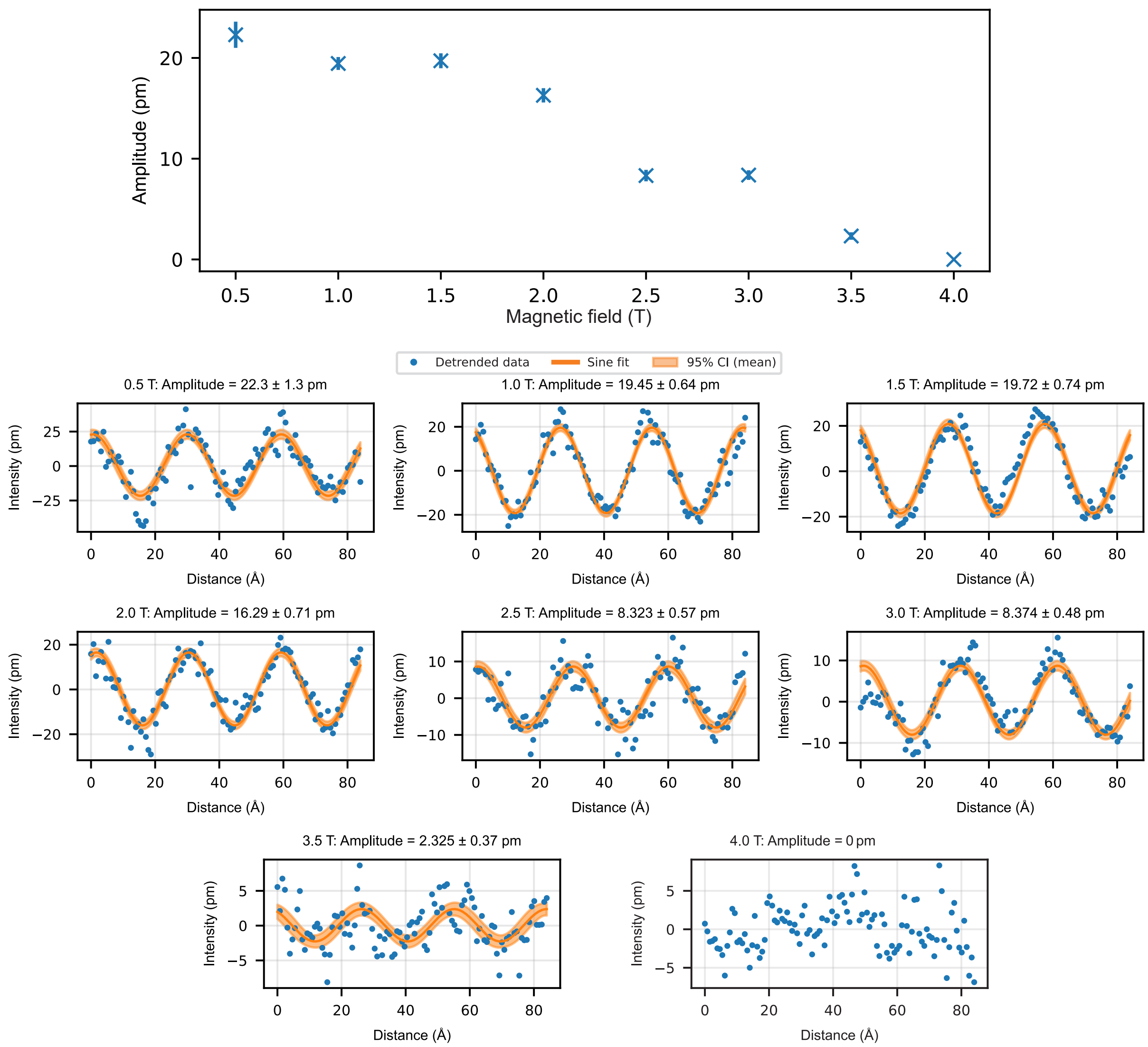

Fig. S5. **Measured stripe amplitude as a function of external magnetic field.** Top: amplitude of the multiferroic stripes extracted from sinusoidal fits to detrended line profiles; the amplitude decreases with field. Error bars are the fit uncertainties. Bottom: detrended line profiles and sine fits at each field.

The line spectra in Fig. S6 show that a polaron shifts the conduction-band (CB) onset upward by more than 400 mV, but that this perturbation is strongly localized, decaying within few nm from the center of the defect. This is comparable to the localized band bending reported for polarons in related monolayer transition-metal halides such as $CoCl_2$[32,33]. Since the multiferroic domains extend over tens of nanometer, each polaron perturbs only a small fraction of a single domain. The two signatures are also spectroscopically distinct: the polaron produces a large shift of the CB onset localized at the defect, whereas the multiferroic stripes produce a much smaller (37 mV) periodic modulation of the CB maximum, with negligible onset shift (main-text Fig. 2d,e). All STS

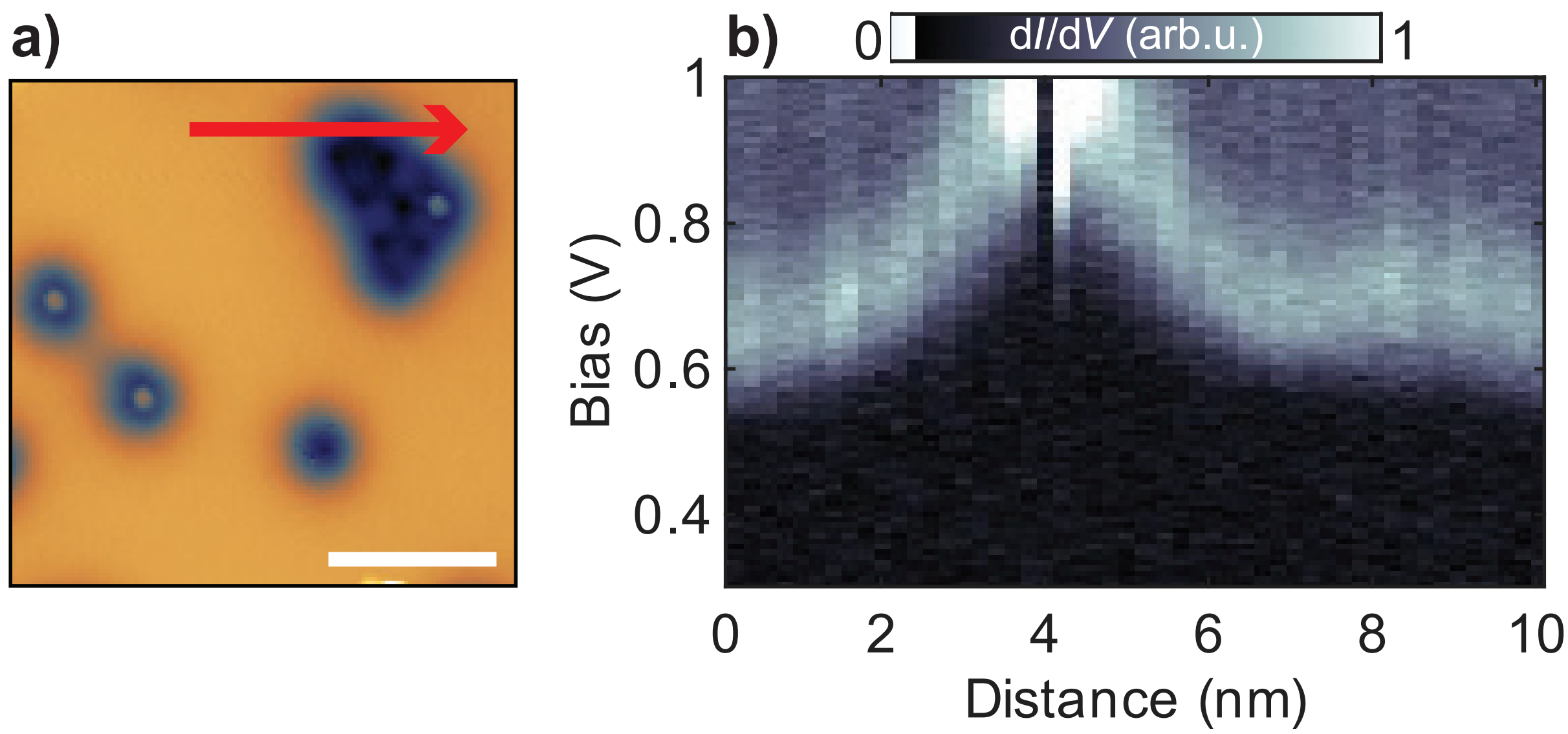

Fig. S6. Investigation of polaron defects on monolayer $NiBr_2$. **a** An atomic scale scan of monolayer $NiBr_2$, with polaron defects clearly visible (1.0 V, 131.1 pA, scale bar 5 nm). Red arrow shows the direction and location of the taken line spectra. **b** A line spectra across a polaron defect. The shift in CB onset is clear.

data reported in the main text were acquired at locations sufficiently far from polaron defects, so the line spectra of Fig. 2d show the smooth periodic modulation of the multiferroic order, without the localized features characteristic of individual defects. Finally, across all measured samples we find no systematic correlation between polaron density and stripe periodicity, nor between polaron motion under bias-pulse manipulation and domain orientation, indicating that the polarons neither drive nor pin the multiferroic order. A detailed study of the polaron properties is beyond the scope of this work.

---

* These authors contributed equally.
